# Do Not Divide Count Data with Count Data; A Story from Pollination Ecology with Implications Beyond

**DOI:** 10.1371/journal.pone.0149129

**Published:** 2016-02-12

**Authors:** Trond Reitan, Anders Nielsen

**Affiliations:** Centre for Ecological and Evolutionary Synthesis (CEES), Department of Biosciences, University of Oslo, P.O. Box 1066 Blindern, NO-0316 Oslo, Norway; Ciudad Universitaria, ARGENTINA

## Abstract

Studies in ecology are often describing observed variations in a certain ecological phenomenon by use of environmental explanatory variables. A common problem is that the numerical nature of the ecological phenomenon does not always fit the assumptions underlying traditional statistical tests. A text book example comes from pollination ecology where flower visits are normally reported as frequencies; number of visits per flower per unit time. Using visitation frequencies in statistical analyses comes with two major caveats: the lack of knowledge on its error distribution and that it does not include all information found in the data; 10 flower visits in 20 flowers is treated the same as recording 100 visits in 200 flowers. We simulated datasets with various “flower visitation distributions” over various numbers of flowers observed (exposure) and with different types of effects inducing variation in the data. The different datasets were then analyzed first with the traditional approach using number of visits per flower and then by using count data models. The analysis of count data gave a much better chance of detecting effects than the traditionally used frequency approach. We conclude that if the data structure, statistical analyses and interpretations of results are mixed up, valuable information can be lost.

## Introduction

Research into applied statistical methodology is a constantly progressing and expanding field giving scientists in other disciplines the opportunity to perform analyzes on increasingly complex datasets. In the urge to reveal patterns and their underlying processes, scientists have used new and advanced statistical methods, often beyond the level of which they are comfortable (e.g. [1]). In scientific disciplines dealing with empirical data, e.g. Ecology, data transformations are commonly used to fit a diverse array of data types to a normal error distribution [2]. The transformed data may fit the assumptions and requirements for the statistical test, but transforming data back and forth may lead to difficulties in the interpretation of results and, as we show here, reduce the statistical power of the test (increased probability of Type II error). Furthermore, improved statistical power means that patterns can be revealed that would not otherwise be detected. Limited statistical power can be counteracted by increased sample size, but in empirical research sampling effort is always a limited resource and insufficient sampling might affect the results of the studies conducted (e.g. [3, 4, 5, 6]). Indeed, studies on sampling completeness, i.e. assessments of how close a sampling regime is to reflect reality, has become a scientific discipline in itself [3, 4, 7, 8, 9]. An important focus for research in applied statistics is therefore to develop methods and frameworks with improved statistical power. Our goal here is to show that dealing with data types in a proper way can improve power to detect important effects, consequently increasing the probability of revealing true patterns within empirical datasets while reducing the minimum sampling effort and resources needed.

We examine models containing several types of effects (fixed categorical, fixed slope, random effect, random slope) commonly encountered in Ecology. We use studies of plant-pollinator interactions as examples of sampling intensive studies where sampling methods [8, 9] and effort [10, 11, 12, 13, 14] is always an issue. However, the results and discussion herein may apply to any field of science that derives frequencies from count data.

Plant-pollinator interactions are ubiquitous in nature, and the focus on how external factors such as habitat fragmentation, pesticide use or climate change might affect plant pollinator interactions is currently high on the research agenda [15, 16, 17]. Studies of plant-pollinator interactions are often plant centered as data are gathered by observing pollinator activity in particular flowers. Most studies investigating how flower visitation varies with respect to external drivers use visitation frequencies, namely number of visits per flower per time. In statistical analysis these frequencies are treated as real numbers with normal distributed errors, or arcsine transformed to better fit a normal distribution (from our own work; [18, 19, 20]. As shown by [2] however, the arcsine transformation is not only statistically wrong, but also exclude important information contained in the data from the analysis and proves unnecessary as modern statistical tools are available to deal with the real distributions found in the data.

Visitation frequencies are derived entities and can be based on one or many actual flower visits (pollination events). For example, observing 100 pollinator visits in 200 flowers over a 15 minute period generate the same visitation frequency as 2 pollinator visits in 2 flowers over a 30 minute period. Both observations generate the frequency of 2 visits per flower per hour; however, the first observation is based on the observation of 100 flower visits, while the second on only 2. If we in parallel had counted flower visits in another 200 flowers over the same 15 minute period, we would had expected considerably less variation than if we were counting flower visits in another 2 flowers over the same 30 minutes period. Thus we would get much more reliable information in the first case than in the second, but by use of visitation frequencies in the analysis this information is lost. When sampling flower visitation data in different study plots, the number of flowers observed (exposure) can be highly variable. These realizations led us to investigate whether it would be feasible to analyze all observed flower visits as separate events (counts) and use a count data distribution instead, for instance using Generalized Linear Models (GLMs; often with Poisson or the negative binomial distribution families) [21]. In such models, the exposure (i.e. number of flowers observed) can be accounted for, so that the full information content of the measurements incorporated into the analysis. This approach should also better fit the underlying statistical process that flower visitation data represent.

We conducted a simulation study under conditions of great variability in exposure, to illustrate the effects of the information loss when using visitation frequencies. With less variability in exposure, the information loss will be less severe. Exposure variations can be driven either by variability in number of flowers observed or in the time used for each measurement, but for simplicity we kept the time constant. Visitation frequency is quantified as number of flower visits per number of flowers per time, the time aspect being ignored for simplicity. Since no distribution sticks out as most suitable for ratios of integers (visitation frequencies), a certain loss in statistical power is to be expected in any case. We take this as yet another argument for analyzing the data as counts rather than the commonly used visitation frequencies (This is further described in the supporting information, section C). In addition to there not being a canonical distribution for this type of data, one would also have to deal with zero-inflation, the extra probability of getting exactly zero pollinators and thus also a frequency equal to zero. This is not only a technical difficulty in an analysis dealing with continuous data distributions, but also reveals a lack of knowledge concerning how the zero-inflation probability will respond to changes in the data distribution.

We simulated a large collection of datasets on flower visits. Each data set either contained a known effect of an imagined external process or lack thereof. We generated datasets with different effect types and count data distributions to assess how external processes of various types affected our results (se “datasets” in the Methods section). The data was then presented in two ways; first as original count data (number of flower visits within a constant time frame with number of flowers as offset) and then as derived visitation frequencies (viz. number of visits per flower unit time).

For all datasets with counts we applied a statistical analysis and counted the number of times data produced without an effect was misclassified as having an effect (type I error) and the number of times data produced with and effect was misclassified as not having an effect (type II error). Another statistical analysis tailored to handling counting rates was then applied on the derived visitation frequencies. A classic analysis would hold the rate of type I errors constant and only report the rate of type II errors. However, because of dealing with non-standard hierarchical models for the visitation frequencies, we opted for a Bayesian analysis which produces a model probability for there being and not being an effect. This probability can then directly be used to classify datasets as showing or not showing an effect, but with the consequence that both the rate of type I and type II errors can vary. Thus in addition to producing a table where both these numbers are presented (for a fixed threshold of the probability), we also create Receiver-Operator-curves (ROC) which show how these two numbers co-vary and thus gives a more direct comparison of how well each method works. Finally we examined how much more data is needed (sampling effort) to detect an effect with a given effect size using visitation frequencies rather than the original count data.

## Materials and Methods

### Data sets

To assess the statistical power of analysis of flower visitation data using visitation frequencies (number of visits per flower per hour; traditional approach) and counts of single visits (new approach) we simulated four types of datasets. The datasets were designed to contain a known distribution and predefined effects of hypothetical external factors (random or fixed effects). For each type of effect we simulated data according to three different distributions. For each combination of effect and distribution, we made 10 000 datasets containing count data and then translated each of these datasets into 10 000 equivalent visitation frequencies datasets. We then built an analysis framework that traversed all datasets, conducted the appropriate statistical tests (see the next section) and summarized the number of occasions where effects were found for the two different data types (counts and frequencies).

As we do not know what distribution nature provides our datasets with, we analyzed our simulated datasets with statistical models assuming several candidate distributions, including a distribution that did not correspond to any of the datasets. Similarly, it is possible that none of the distributions in our statistical models describes the distribution provided by nature. We therefore generated datasets using several distributions, including a distribution not represented in our analytical models. This type of setup allowed us to test the robustness of the various analytical methods under contrasting violations of the model assumptions. In summary, we made a point of having one distribution in the datasets that was not represented in the analysis, and one distribution represented in the analysis that was not present in the simulations.

The effects simulated in the datasets were assumed to change the expected visitation frequency (per flower), *λ*, thus all distributions had *λ* as one of their parameters. In our interpretation of the analyses, we always used the visitation frequency as the focal variable as it is easy interpretable. Nuisance parameters (if any) were assumed to be unaffected by the main effects. It was assumed that for each possible effect, the nature of that effect would be known in advance. That is, there would be no confusion as to whether a covariate was categorical or continuous, random or fixed. All effects, continuous or categorical, fixed or random, were examined using a log-link function. That is, the log-transformation of *λ* and then the application of a standard linear model on that. This means that for a given covariate x (categorical or continuous), *log*(*λ*) = log(*λ*_0_) + *βx* for a fixed effect and *log*(*λ*) = log(*λ*_0_) + *ε*_*x*_ for a random effect. *λ*_0_ was set so that the expected number of pollinators per flower was 0.02 when *x* = 0. The particular number for the base expected visitation rate, *λ*_0_, was chosen as a compromise between two opposing considerations. Firstly we wanted a sizeable percentage of the simulated measurements to result in zero counts even with the large spread in in the number of flowers (see the later description of how we sampled that). With the chosen *λ*_0_ and the chosen sampling scheme for the number of flowers, we got about 12.7% zeros. Secondly, we wanted a flower visitation rate that could be considered realistic. Our previous experience [20] indicated that visitation rates for various pollinator groups could vary at least between 0.05 and 1.0 for that particular study when using a 10 minute observation period. We went a little below that range in order to get more zero-observation. Time spent observing was kept constant to ease the construction of the data sets and the interpretation of the results. This is justified by the fact that observation time is easier to standardize than the number of flowers in real studies. The effects introduced to the datasets were as follows:

**1) Categorical (binary) fixed effect.** This represents a covariate that can be in one of only two states, which thus can represent a single yes/no (1 or 0) process. The expected visitation frequency will be different for the two states. For each dataset, we opted for sampling the categorical covariate with 40% probability for “no” and 60% probability for “yes”. Categorical fixed effects might represent a test for differences in flower visitation between flowers growing in a wild system and in an agricultural setting, flowers experiencing pesticides or not, or crop flowers grown inside and outside growing tunnels. Mathematically, a binary effect can be summarized as *λ*(*x*) = *λ*_0_ if *x* = 0 and *λ*(*x*) = *λ*_1_ if *x* = 1, where *x* is the binary covariate.

**2) Continuous fixed effect.** Here, the log-transform of the expected visitation frequency is assumed to change linearly along a continuous covariate (which was sampled from a uniform distribution). This scenario might represent a test for how flower visitation is varying along an environmental gradient, e.g. elevation or latitude. It could also represent a temperature response, a temporal change throughout the season or a response to increasing levels of pesticides. Mathematically, a continuous fixed effect can be summarized as *λ*(*x*) = *λ*_0_*e*^*βx*^ for a simple linear effect on log-transformed visitation frequency (though more complicated formulas could also be used).

**3) Random intercept (categorical random effect).** This is similar to the categorical fixed effect, except that the expected visitation frequencies of the different categories are (effectively) drawn from a random distribution. We chose to use five categories rather than two (as for the fixed effects), since it was less probable that five independent samples would be virtually the same than that two such samples would be so. For each dataset, these categories were traversed systematically. A random intercept might represent five different farms or five different regions. Mathematically, a random intercept can be summarizes as $\lambda \left(x\right)=\lambda_{0}e^{\varepsilon_{x}}$ where the random effect $\varepsilon_{x}∼N(0,\sigma_{RE}^{2})$ is drawn randomly for each category, *x*.

4) Random slope (*continuous random effect)*. Here there are two covariates, one categorical and one continuous. The logarithm of the expected visitation frequency is assumed to depend linearly on the continuous covariate. However, the slope is assumed to be drawn randomly from a distribution for each value the categorical covariate can take. As for random intercept, we used five categories and traversed each combination of categorical and continuous covariate value systematically. Random slope illustrates how an ecological process might show contrasting effect in different areas, e.g. that increased temperature might increase flower visitation in some areas but reduce or have no effect on it in others. Mathematically, a random slope can be summarized as $\lambda \left(x,y\right)={\lambda_{0}e}^{\beta_{x}y}$ where *x* is the category; *y* is the continuous value and $\beta_{x}∼N(0,\sigma_{RS}^{2})$ is the random slope, one for each category.

When studying random effects (random intercept or random slope) it turned out to be important to at least traverse the covariate values systematically and to have enough data per categorical state to be able to detect the distribution of the random effects. With the effect pronounced enough, just 30 data points per dataset was sufficient for studying fixed effects (case 1 and 2) in a reliable fashion. However, since random effects could more easily end up with slight differences due to stochasticity, we opted for 75 data points per dataset so 15 data points per category (for instance farm) for random effects (case 3) and 300 data points so 60 data points per category for random slope (case 4), in order for these effects to more reliably be detected.

There are several available distributions for describing count data. As we do not know what we might encounter in nature, several distributions were used for generating the datasets to be analyzed. In this way, we were able test how well we could detect effects when the count data had been produced according to several scenarios. The following three distributions were used in the data simulations: The Poisson distribution (for counts of independent events), the negative binomial distribution (an over-dispersed distribution frequently used in analysis), the lognormal-Poisson distribution (another over-dispersed distribution which is less used in analysis). The distributions are further described in the supplementary information, section A.

When generating each dataset we constructed each row (observation event) independently. First, the number of flowers observed was sampled from a negative binomial distribution, adjusted so that there was a 95% probability of the number of flowers being larger than or equal to 10 and smaller than or equal to 1000 (Measurements with zero flowers were re-sampled). Second, the covariate value was chosen (see the description of each effect). Finally, the number of bees was sampled according to the chosen distribution, the number of flowers and the covariate value.

We simulated datasets both with and without an effect, so that we could check the probability of detecting effects not present in the data (type I error) and the probability of not detecting effects present in the data (type II error). For all effects, we made 10000 datasets. Thus in total, we simulated 4 (effect types) x 3 (distributions) x 2 (effect/no effect) x 2 (count/frequency) x10000 = 480 000 datasets.

For each combination of effect and distribution, the strength of the effect (the difference in λ(x)) for different values of the covariate value was adjusted so as to produce approximately 10% false negatives (i.e. a type II error of 0.1) for the analysis of count data. This is to ensure that the test results of the two analytical approaches were not obvious and that the probability of not detecting an effect, if present, was real.

Lastly, for binary fixed effects, we also experimented with the sampling size of the visitation frequency data (holding the effect strength constant), in order to reproduce the approximately 10% false negative rate we aimed for in the count data analysis. This experiment was performed in order to see how much more data would be needed for the frequency data in order to get the same statistical power as count data analysis.

### Analysis

The objective of the analysis was to compared the percentage of false negatives for the simulated visitation frequency datasets to the ~10% level given for the corresponding count datasets, thus giving a summary of the relative statistical strength of using count data versus frequency data. In the same way, differences in false positives (i.e. Type I error) rates were produced by counting the number of datasets for which the analysis falsely indicated a positive result (effect found) when no effect was present.

When analyzing the simulated datasets, we assumed three different possible distributions for the count data: the Poisson distribution, the negative binomial distribution or the zero-inflated negative binomial distribution. We assumed zero-inflation to be a remote possibility in real life count data, at least in our case, but included it in the analysis as an example of a model used in the analysis but not represented in the data. To analyze visitation frequency data, we used models developed by Nielsen *et al*. [20]. As the observed frequencies are such that either positive real values or 0 with a finite probability for this specific outcome are sampled, we were seeking a distribution that had this property. In addition we wanted our model to be built so that the probability of zero-inflation goes up as the expected number of visits goes down (either due to lowered flower visitation frequency or reduced exposure). However, we cannot be *á priori* sure how strong this connection is. Thus an adjustable link between the expected value of the continuous distribution and the zero-inflation probability was also needed. In Nielsen *et al*. [20], we decided on using a gamma distribution with a zero-inflation probability linked to the expectancy, μ, of the gamma distribution by *P*(*x* = 0) = *p*_0_ = *e*^*g*^ *μ*/(1 + *e*^*g*^ *μ*), where *g* is a parameter that controls how the zero-inflation responds to changes in the expectancy. This is also explored in section C of the Supporting Information. Effects were incorporated so that the expectancy value of the distribution could change due to a change in a covariate value. The nature of the effect, if there was any (fixed categorical, fixed linear, random interceptor random slope), was assumed known in advance. The analysis of count data would then proceed by analyzing six models, namely Poisson, negative binomial and zero-inflated negative binomial all three with and without an effect. For the frequency data we analyzed one non-effect model and two effect models, one where the effect only showed itself directly on the expectancy of the gamma distribution and one where it also directly affected the zero-inflation (see [20], Supporting Information, section B).

The best model for each dataset was determined using Bayesian model likelihoods (BML, see below). We then counted the number of datasets which were classified as having an effect and number of datasets not having an effect. This was done both for datasets made up of derived frequency data and for datasets containing the original count data, with number of flowers as an offset variable. We included all datasets; those were an effect was present and those where it was not. When analyzing datasets where there was no effect, the objective was to count the number of false positives and, when data had been simulated with an effect, the objective was to count the number of false negatives.

Our analysis involved non-standard models (in particular the zero-inflated gamma distribution) and such models might need further refinement to be used in real-life applications. No readily available software was available for examining such models. We therefore adopted a Bayesian analytic framework following [20]. Furthermore, ecological data often comes with some prior knowledge on possible values they may take, an advantage that can be utilized in Bayesian statistics only. We used an MCMC algorithm to estimate each model. An importance sampling method (developed by Reitan & Pedersen-Øverleir [22]) was then used for estimating the BML. BML determines the Bayesian posterior model probability, $P(M|D)=\frac{P(D|M)P(M)}{\sum_{i=1}^{m}P(D|M_{i})P(M_{i})}$ where D is data, M is model (one among a total of m models), *P*(*D*|*M*) is the BML, *P*(*M*) is the prior model probability and *P*(*M* | *D*) is the posterior model probability [23]. Thus we used BML similar to how one would use a model selection criterion, selecting the model with the highest BML (assuming equal prior model probability). For count data, we had 6 models namely Poisson, negative binomial and zero-inflated negative binomial distribution with and without an effect. If the model with the highest BML was among the effect model, the result of the analysis would be counted as an effect having been found. Wide but informative prior distributions were used on all occasions for all parameters, e.g. Supporting Information, section B.

## Results

We found an increase in the number of occasions where we detected effects in our simulated datasets when conducting analysis on count data, using number of flowers observed as an offset variable. For instance, when using Poisson distributed count data affected by a binary fixed effect the number of false negative (rejection of the hypothesis that there is an effect, when in fact there is an effect) was reduced with more than 50%. The number of instances of false rejection from using the two analytical approaches in increasingly complex models is presented in Table 1.

**Table 1:** False negative rate (type II error, upper panel) and false positive rate (type I error, lower panel) for the analyses of 10000 simulated datasets in each case. The datasets were generated by use of three different distributions (Poisson, Negative binomial and lognormal Poisson). The best model (Poisson, negative binomial and zero-inflated negative binomial for count data) with and without effect was identified and compared to each other using BML. Datasets with an effect were simulated so as to yield approximately 10% false negative for the count data analysis. Each cell contains the percentage of false negatives/positives for the data type (count of frequency) followed by the ratio between them (frequency score divided by count data score). Note that a ratio >1 is favoring the count data approach while ratio < 1 is favoring the frequency approach. These are indicated in bold. Ratios larger than 4 in favor of count data is indicated with bold italic characters.

Effect type	Distribution								
	Poisson			Neg. binomial (k = 10)			Lognormal-Poisson (sd = 0.36)		
**False negative**	**Count**	**Freq.**	**Ratio**	**Count**	**Freq.**	**Ratio**	**Count**	**Freq.**	**Ratio**
Fixed categorical	13.2%	34.8%	2.6	10.2%	24.2%	2.4	10.4%	23.2%	2.2
Fixed linear	9.9%	40.9%	***4*.*1***	9.0%	21.4%	2.4	9.4%	19.6%	2.1
Random intercept	6.1%	19.6%	3.2	5.0%	10.8%	2.2	4.3%	7.7%	1.8
Random slope	9.0%	22.0%	2.4	10.6%	8.4%	**0.8**	10.2%	7.2%	**0.7**
**False positive**									
Fixed categorical	0.62%	3.0%	***4*.*8***	1.2%	3.2%	2.7	1.5%	3.3%	3.2
Fixed linear	1.8%	4.9%	2.7	3.8%	5.2%	1.4	4.1%	5.6%	1.4
Random intercept	2.0%	2.7%	1.4	1.9%	2.2%	1.2	2.0%	2.7%	1.4
Random slope	1.5%	11.1%	***7*.*4***	1.5%	9.5%	***6*.*3***	1.7%	7.7%	***4*.*5***

As can be seen, for most types of effect and most simulation distributions, the advantage in using count data rather than frequency data comes both in the form of lower false negative and false positive rates. For random slope, the over-dispersed distributions seem to yield slightly worse results for detecting effects for count data than for frequency data. However, the false positive rates are much lower for frequency data that for count data in this case (Table 1). In frequentist terms, that means that we are comparing detection rates for two methods of vastly different confidence levels. There is, in most cases, reason to expect increased statistical power from analyses of the real count data as compared to analyses of derived frequency data. However, it is still possible for any one dataset to produce a true positive using frequency analysis but not in the count data analysis. For instance, for a fixed linear effect with Poisson distribution, count data analysis incorrectly indicated no effect in 9.9% of the cases while frequency data analysis produced 40.9% false negatives. Still we found effect in 1.4% of all datasets using frequency analysis but not in the count data analysis. In this case, both analyses failed together in 8.5% of the datasets while in 32.5% of the datasets the frequency data analysis failed while the count data analysis succeeded (Table 1). Our analysis suggest that the analysis of frequency data can succeed in finding an effect while the count data fail, but that this scenario is far less likely than the opposite case.

**Fig 1:**
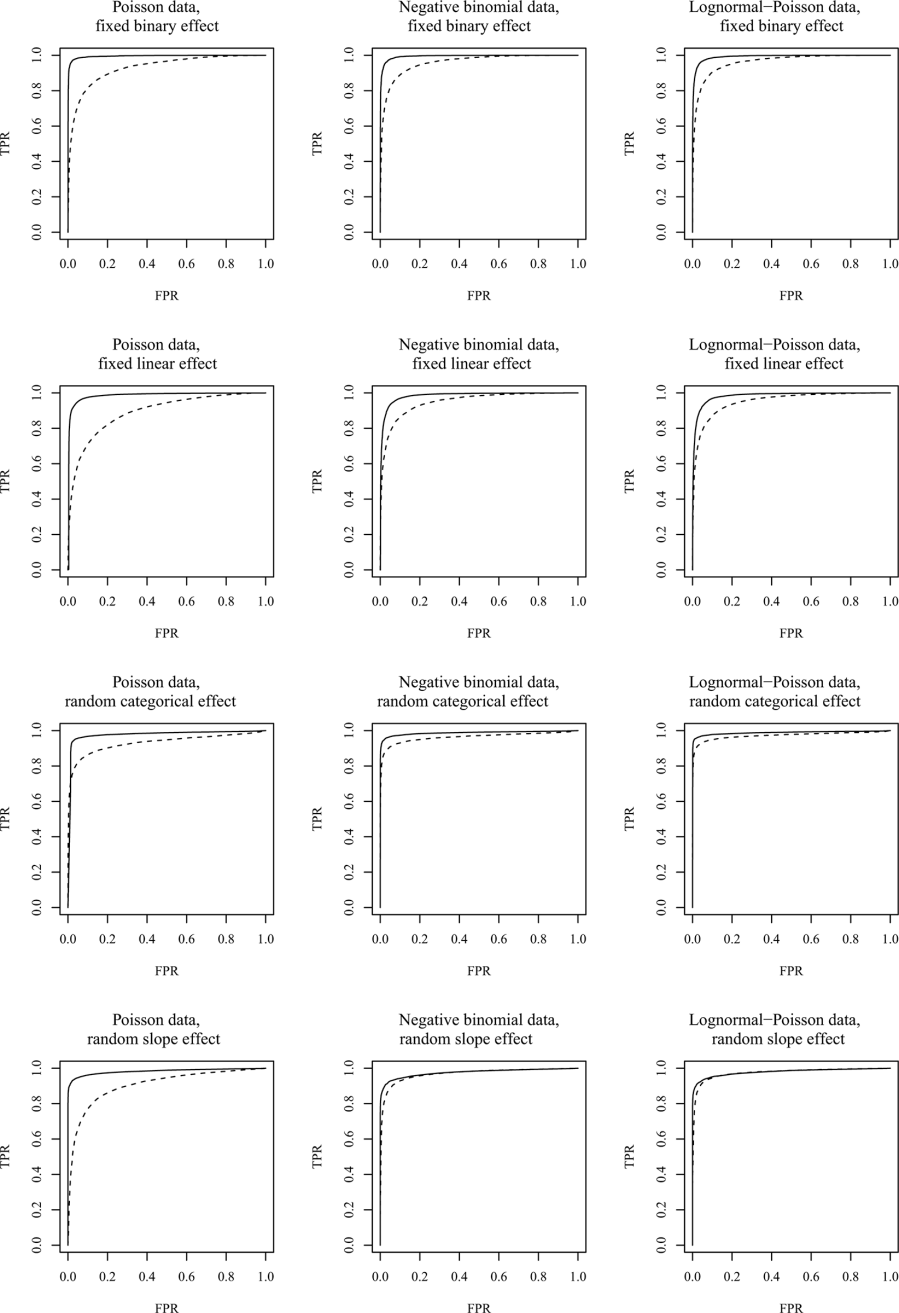
ROC curves (false positive rate against true positive rate) for the different combinations of effect type and data distribution. Solid and dashed lines illustrate the relationship for count data and frequency data respectively.

One problem with a tabulation of Bayesian model choice outcomes is that one does not use a method with a fixed false positive rate. When both the false positive and false negative rate differs in opposite directions between methods, it can be challenging to asses which is the best (e.g. the random intercept and the random slope negative binomial models in Table 1). To more directly compare the strength and confidence between tests, we created ROC curves by increasing the sensitivity of the analysis and plot the true positive rate (strength) as a function of the false positive rate (which when fixed will be the test significance level = 100%-confidence). Thus it shows test strength as a function of 100%-confidence level for a variation of confidence levels in a classic testing scheme. A variation in the sensitivity can be achieved by varying the prior model probability for effect and no effect. This is equivalent to varying the threshold for how large BML_effect_ / BML_no effect_ needs to be for an effect to be declared found. Equal prior model probability between effect and no effect is achieved with threshold = 1. The ROC curves for the different combinations of effects and distributions are shown in Fig 1. Not surprisingly, all combinations of data and model distributions yield better ROC curves (the line falls above and to the left of then other) for count data than for frequencies.

The area under the ROC curve (AUC) gives a quick summary of how well a method for distinguishing effects from the lack of effect works: it gives the probability for a test to rank a randomly chosen dataset with an effect higher than a randomly chosen dataset without an effect, whether there is an effect or not. AUCs for our different analytical approaches and simulated datasets with different effect types are summarized in Table 2. As can be seen from the table, the AUC is always better for the count data analysis than for the frequency data analysis. Quite often, the remaining area over the graph for count data is smaller than the difference between count data and frequency data, thus more than doubling the probability for correctly assigning effect/non-effect to two random datasets.

**Table 2:** Area under curve (AUC) for the different combinations of effects and distributions. In the analysis, the best model (Poisson, negative binomial and zero-inflated negative binomial for count data) with and without effect was identified and compared to each other using BML. Delta AUC is also given to illustrate the difference between the two models utilizing the two different data types (counts and frequencies). Note that the analyses using count data always perform better than the analyses using frequency data, though the difference is, in some cases marginal, e.g. random slope models drawn from datasets built on negative binomial or lognormal Poisson distribution.

Effect type	Distribution					
	Poisson		Neg. binomial (k = 10)		*Lognormal-*Poisson (sd = 0.36)	
	Count	Freq.	Count	Freq.	Count	Freq.
Fixed categorical	99.5%	92.9%	99.5%	95.9%	99.2%	96.3%
Fixed linear	98.6%	89.1%	98.4%	95.0%	98.3%	95.3%
Random intercept	97.6%	93.1%	98.7%	96.3%	98.8%	97.2%
Random slope	98.3%	90.4%	97.6%	96.8%	97.9%	97.5%

Finally, we examined how much the number of data points in the visitation frequency data had to be increased in order to replicate the statistical power of the count data (≈10% false negative rate). This was done for one particular combination of effect and distribution, namely a fixed binary effect with Poisson distributed data. The experiment was computationally costly, since it involved a trial and error search for the number of data points in the visitation frequency data. Thus we did not perform this experiment for all combinations of effect and distribution.

In that particular experiment, we found that we needed to go from 30 data points to 60 per dataset to get approximately 10% false negatives for the given effect. As can be seen in Table 1, this combination of effect and distribution is not unique in terms of difference in statistical strength for count data and visitation frequency data.

## Discussion

We have shown that analyses using original count data and models assuming count data error distribution (e.g. Poisson) are more likely to yield correct results as compared to analyses of derived frequency data assuming a normal error distribution on transformed data. How much is gained by analyzing count data rather than frequency data depend on the variability in the exposure, the effect type and strength and the underlying distribution of the simulated data. In some cases, working with count data can make it more than 4 times more likely that an effect is revealed than when working with frequency data (for fixed linear Poisson data; Table 1). We found that different models and distributions gave varying degrees of advantage for the count data approach, which suggests that for some combinations of effect type, effect strength, data size and distribution, the gain may be even larger. We acknowledge that sample size and effect strength affect how well the different methods perform, but we do not expect the ranking of the two modelling approaches to change. This is something we experienced in the initial phase of the analysis of each effect, when we were varying the strength of the effect in order to approach the target false positive rate of about 10%.

Several studies have highlighted the importance of using proper distributions in statistical analyses and that violations regarding the underlying assumptions for the tests conducted might cause bias in the results [24] and/or create hard to interpret or even nonsensical predictions [2]. We add to the list of arguments that proper statistical modelling will improve the strength of the statistical tests, increasing the probability of detecting true patterns within the data consequently reducing the sampling effort needed to reveal the focal patterns.

The main reason for count data outperforming derived frequencies in statistical analyses is simply that count data contain more information (the exposure), which must be expected to aid inference. In our example, the flower visits are the events and the number of flowers observed is the exposure, included in our models as an offset variable. Frequency data on the other hand compile the number of flower visits and the number of flowers into one number. This number might be the same for two observation events but still it can be based on a very different number of observed flowers and flower visitors. This might be similar to species distribution modelling, where it turns out that count data leads to more accurate predictions than if the data is collapsed into presence-absence [25].

Method strength translates directly into how much data is needed in order to reveal an effect that is present within the dataset. For our example with Poisson distributed data with a binary covariate, we found that for frequency data we needed to double the sample size in order to get the same true positive rate as for count data. If cost of data sampling scales linearly, this means that it will cost twice as much to gather sufficient data to reveal an effect, if one choose to analyze the data as frequencies instead of as counts. Or in other words; that one will produce only half the number of detections of new effects structuring the dataset under a frequency data regime than under a count data regime.

Here we have focused on analyses of plant-pollinator interactions as these types of data we are particularly familiar with. However, we expect any study dealing with count data as outcome and with varying exposure to yield similar results. The choice of parameters included here might be adapted to plant-pollinator interactions but the statistical structure behind will be the same. Thus studies involving the density of animals per area, flowers of a given species per meadow or parasites per host area will all benefit from an analysis that works with count data and exposure. Data where these two have already been aggregated into one frequency variable has been intensively used. In parasitology however, statistical analyses using a count data approach is often preferred over frequency approaches [26, 27, 28]. As with pollination ecology, count data probability distributions are regarded as part of the underlying ecology of parasite population dynamics, and yield a more correct description of the system under investigation [29, 30]. The main reasons for this, we believe, are historical, namely that not so long ago statistical software could not take these types of models into account, but also because frequencies are easily interpretable. Number of visits per flower per hour is an easy concept to grasp and compare among sites or species or along environmental gradients. All modern statistical software can handle count data with Poisson (or even more sophisticated distributions) and technically these are not different from classical linear regression approaches when implemented in e.g. R (in for instance the packages lme4 [31] or glmmADMB [32,33]). With respect to interpretability, we emphasize the importance of separating between that which is to be inferred and the structure of the data used for this inference. The input to the analytical tools is based on count data distributions, while the output is a mean number of visits (which may not actually be a frequency). However, with an offset, the output is directly interpretable as rates, i.e. the expected number of visits per flower, which is precisely the quantity of interest. If the data structure, statistical analyses and interpretations of results are mixed up, valuable information can get lost, as we have shown here.

## Supporting Information

S1 Fig Histogram of ratios, z = x/y, where x and y are sampled from the negative binomial distribution. The total count for z = 0 is not shown as it is much higher than the other ratios.
(EPS)

S2 Fig Kernel smoothed density functions for ratio samples and a range of smoothing window size options (using the "density" function in R). Solid line: adjustment = 1, short dashed line: adjustment = 2.5, long dashed line: adjustment = 10.
(EPS)

S1 Text Bayesian model inference, the distribution of a ratio of count data, project code. (DOCX)
