# Supplementary material for: Do Not Divide Count Data with Count Data; A Story from Pollination Ecology with Implications Beyond
Source: PLoS One. 2016 Feb 12;11(2):e0149129. doi: 10.1371/journal.pone.0149129 (PMC4752487; doi:10.1371/journal.pone.0149129)
Supplement: S1 Text — (DOCX) [file pone.0149129.s003.docx]

**S1 Text: Bayesian model inference, the distribution of a ratio of count data, project code.**

Trond Reitan* & Anders Nielsen

*Centre for Ecological and Evolutionary Synthesis (CEES), Department of Biology, University of Oslo, P.O. Box 1066 Blindern, NO-0316 Oslo, Norway*

* Corresponding author email: trond.reitan@ibv.uio.no

### Supporting Information, section A – distributions used for data generation

A) The Poisson distribution; $P\left( k \right)=\frac{\left( N\lambda\right)^{k}}{k!}e^{-N\lambda}$. This distribution describes the counting of independent events (e.g. number of flower visits), *k*, for a given exposure (the number of flowers), *N*, and a given expectancy per exposure, *λ*. Taking the exposure into account is often described as using an offset in model fitting. Since the Poisson distribution only has one parameter, the variance as well as the expectancy is determined by this parameter, E(*k*)=Var(*k*)=*Nλ*.

B) The negative binomial distribution; $P\left( k \right)=\left( \begin{aligned} k+r-1 \\ k \end{aligned} \right)\left( \frac{r}{N\lambda+r} \right)^{r}\left( \frac{N\lambda}{N\lambda+r} \right)^{k}$. The quantities *k, N* and *λ* have the same interpretation as for the Poisson distribution*.* In addition, there is a shape parameter, *r,* determining the over-dispersion of the distribution, $Var\left( x \right)=E\left( x \right)+{E(X)}^{2}/r$. If *r* is very large, the negative binomial distribution approaches the Poisson distribution, while for low values of *r*, the difference (and over-dispersion) will be large. Note that the distribution has been parametrized so that the expectancy per exposure is one of the parameters. This distribution can be generated by sampling from the Poisson distribution with exposure *N* as before and a random Poisson parameter which is gamma-distribution with expectancy *λ* and shape parameter *r*. Thus it can represent a count where there are unmeasured random (gamma distributed) effects that affect the frequency independently for each measurement. We chose *r*=10, so as to get a distribution noticeably different from the Poisson distribution.

C) The lognormal-Poisson distribution; $\left( k \right)=\int_{0}^{\infty} \frac{1}{\sqrt{2\pi}\sigma\Lambda}e^{\frac{-\left( log\left( \Lambda\right)-log\left( \lambda\right) \right)^{2}}{2\sigma^{2}}}\frac{\left( N\Lambda\right)^{k}e^{-N\Lambda}}{k!}d\Lambda$ .

The quantities k, N and λ have the same interpretation as for the Poisson distribution, while σ is the variance of the log-normal component of the model and controls the degree of over-dispersion (similar to r in the negative binomial distribution). This is an over-dispersed distribution, constructed in the same way as the negative binomial distribution except that a the random Poisson parameter ** is drawn from the log-normal distribution, so that $log\left( \Lambda\right)\sim N\left( log\left( \lambda\right),\sigma^{2} \right)$. Since we consider measured random effects as normally distributed corrections to $log\left( \lambda\right)$, this distribution represent the same treatment for unmeasured random effects. It has been used in an ecological context in parasitology, see e.g. [21]. While we used this distribution for making simulated datasets, the model was not used as an optional distribution in the analysis. This in order to test how well the analysis performed when the data is distributed differently than any of the distributions considered in the analysis.

### Supporting Information, section B – Bayesian model inference

Bayesian model inference is based on Bayes formula, which gives a posterior probability for parameters and models given data and a prior distribution. This looks like Eq. (A1) for model inference and like Eq. (A2) for parameter inference for a given model:

$\Pr\left( M | D \right)=\frac{P\left( D | M \right)P(M)}{\sum_{i=1}^{m} P\left( D | M_{i} \right)P(M_{i})}=\frac{P\left( D | M \right)P(M)}{P(D)}$ (A1)

$f\left( \theta| D,M \right)=\frac{f\left( D | \theta,M \right)f(\theta|M)}{\int f\left( D | \theta,M \right)f\left( \theta| M \right)d\theta}=\frac{f\left( D | \theta,M \right)f(\theta|M)}{f(D|M)}$ (A2)

Here $M$ is one among $m$ models, $M_{1},\cdots,M_{m}$, $D$ is the data and $\theta$ is the parameters of model M. $f\left( \theta| D,M \right)$ is the posterior distribution, $f\left( \theta| M \right)$ is the prior distribution and $f\left( D | \theta,M \right)$ is the likelihood function for that particular model. Similarly $\Pr\left( M | D \right)$ is the posterior model probability, $\Pr(M)$ is the prior model probability and $P\left( D | M \right)$ is the Bayesian model likelihood, which we abbreviate to BML here.

The prior distribution of the parameters were chosen wide but informative, so as to facilitate model comparison. We kept each parameter independent from the others, since we did not have any prior information concerning connections between them and in order to make the specification of the prior as simple as possible. We assigned a normal distribution on transformed parameters, so as to keep the possible parameter values within their possible values. Thus for count data parameter models, we log-transformed the base flower visitation frequency, $\lambda_{0}$, the random effect/random slope standard deviations $\sigma_{RE}/\sigma_{RS}$, the shape parameter of the negative binomial distribution, $r$, and logit-transformed (log(x/(1-x)) the zero-inflation probability, $p_{0}$. Fixed effect itself, $\beta$, would be untransformed. (So also would random effect, but in this case their distribution would be determined by parameters, not by a prior distribution). The normal distributions were chosen so as to meet a target 95% credibility interval for each parameter on the original scale. These 95% CIs were $\lambda_{0}\in\left( 0.009,110 \right), \beta\in\left( -9.8,9.8 \right),r\in\left( 0.009,110 \right), \sigma_{RE}\in\left( 1.5*{10}^{-8},6.8*{10}^{7} \right), \sigma_{RS}\in\left( \frac{1}{18000},18000 \right), p_{0}\in\left( 0.0004,0.9996 \right)$.

The frequency data distribution used for analysis was a zero-inflated gamma distribution (see also Nielsen et. al 2012):

$$f\left( z|x \right)=p_{0}\left( x \right)\delta(z)+(1-p_{0}\left( x \right))\frac{{\mu(x)}^{\alpha}}{\Gamma(\alpha)}z^{\alpha-1}e^{-\mu(x)z}$$

where *z* is the frequency, x is the covariate (or *x* and *y* in the case of random slopes, where *x* is the categorical effect and *y* is the gradual covariate) and$\delta(z)$ is Dirac’s delta distribution, which assigns all probability to *z*=0. The null model will have $\mu\left( x \right)=\mu_{0}$ and $p_{0}\left( x \right)=\frac{g\mu}{1+g\mu}$ so as to assure that $0<p_{0}\left( x \right)<1$. Model one will modify $\mu\left( x \right)$ according to *x*, but the zero inflation only changes due to the changes in $\mu\left( x \right)$, so that $p_{0}\left( x \right)=\frac{g\mu(x)}{1+g\mu(x)}$. For a fixed slope analysis and for a fixed binary analysis, $\mu\left( x \right)=\mu_{0}e^{\beta x}$, for a random categorical effect analysis, $\mu\left( x \right)=\mu_{0}e^{\varepsilon_{x}}$ where $\varepsilon_{x}\sim N(0,\sigma_{RE,\mu})$ and for a random slope analysis $\mu\left( x,y \right)=\mu_{0}e^{\varepsilon_{x}y}$ where $\varepsilon_{x}\sim N(0,\sigma_{RS,\mu})$. Model two had the same treatment for $\mu\left( x \right)$, but in addition the zero-inflation connection parameter *g* could change with changing covariate values. Thus for fixed slope analysis and for fixed binary effect analysis, $g\left( x \right)=g_{0}e^{\beta x}$, for a random categorical effect analysis, $g\left( x \right)=g_{0}e^{\eta_{x}}$ where $\eta_{x}\sim N(0,\sigma_{RE,g})$ and for a random slope analysis $g\left( x,y \right)=g_{0}e^{\eta_{x}y}$ where $\eta_{x}\sim N(0,\sigma_{RS,g})$.

The location parameter $\mu_{0}$, the shape parameter, $\alpha$, and the zero-inflation connection parameter , $g$, and the random effect or random slope standard deviations would be log-transformed before being assigned a normal distribution, while as before $\beta$ was not transformed. The 95% CIs were $\mu_{0}\in\left( 0.009,110 \right), g_{0}\in\left( 0.0001,10000 \right), \beta\in\left( -10,10 \right), \alpha\in\left( 0.0001,10000 \right),\sigma_{RE,\mu/g}\in\left( 1.5*{10}^{-8},6.8*{10}^{7} \right), \sigma_{RS,\mu/g}\in\left( \frac{1}{18000},18000 \right)$.

For many models, including most of those used here, Eq. (A2) cannot be solved analytically. The reason is that the integral expressing the BML can in such cases not be calculated analytically. Thus parameter estimation must rely on numerical methods, such as Markov chain Monte Carlo (MCMC) sampling. With MCMC sampling, one proposes a new parameter value given the previous sample (thus the Markov chain aspect), and accept that proposal with a probability given by the Metropolis-Hasting algorithm (see for instance Chib & Greenberg 2001 or Hastings 1970). In our case, we used a very simple MCMC sampling scheme known as random walk Metropolis sampling (Metropolis 1953), though we adjusted the random walk jumps so as to get a target acceptance rate of 1/3 in the burn-in phase. We also used parallel tempering (Geyer 1991) to deal with the possibility of multimodality in the posterior parameter distribution.

As seen in Eq. (A1), the BML is needed for calculating the posterior model probability. If the models are assumed á priori equally plausible, the BML or log-BML can be used directly to single out the best model given the data (and the prior). Model likelihoods will not necessarily always increase for increasing model complexity, but will rather support a simpler model if the data is sufficiently described by it. Thus parsimony is a factor in BML-based model choice, see for instance Jefferys & Berger (1992). As mentioned, BML is often not analytically available. In this study we used an importance sampling method for estimating the BML described in Reitan & Petersen-Øverleir (2009).

### Supporting Information, section C – The distribution of a ratio of count data.

The visitation frequency in dataset is formed by taking the ratio of the number of pollinators over the number of flowers (exposure). As mentioned in the main text, we do not know of any readily available distribution for such data. We will here study this distribution by simulated data. We drew one million samples of exposures, $y\sim nbinom(\lambda=5,r=1)$, thus a fairly over-dispersed exposure distribution. Samples where *y=0* where re-sampled. We then sampled the numerator (#pollinators) from $x\sim nbinom(\lambda=0.5y,r=1)$ for each sampled *y*. Thus our simulation is so that the expected visitation frequency is 0.5. We then form the ratio, $z=x/y$ for these one million samples. With that many samples, the histogram from these samples should be a good approximation for the actual distribution. The histogram is shown in figure S1, and indicates the problem with assigning probability density functions for such random variables. The histogram is not continuous but contains a set of spikes corresponding to certain frequently occurring rational numbers. The simplest rational numbers (0,1,1/2,3/2,1/3 etc.) seems to be the most frequent. Different negative binomial parameters or other count data distributions might alter the relative probability of these ratios, but the general pattern can be expected to remain.

A probability density function cannot be assigned, since the distribution appears by sampling over two discrete probability distributions, one for *x* and one for *y*. In addition, the rational numbers are countable, just as the natural numbers, so a probability for each outcome can be assigned instead of a probability density function:

$P\left( Z=z \right)=\sum_{x,y|\frac{x}{y}=z} P\left( X=x \right)P(Y=y)$ (A3)

where X,Y and Z are the random variables taking values *x,y* and *z* respectively. Thus ideally, this would mean having a model for both the numerator, *x*, and the denominator *y*. This again would mean having to model both the unknown numerator and denominator and then dealing with equation A3, as well as having to deal with numerical errors in the data due to a finite number of digits. Thus such an approach seems hardly feasible for inferential purposes.

A kernel smoother can be used in order to get a probability density function from the samples. (This would again not be feasible in an inferential setting, since there the original sampling distribution is not known.) The result of this using different smoothing window sizes is shown in figure S2. (The “density” function in R with a variation in adjustment settings was used for this purpose). A small smoothing window will relatively accurately follow the target distribution, but as can be seen in the figure, this results in a very irregular probability density function. The smoothing window must be set very high in order to get a function that looks regular, but the problem with this is twofold. Firstly, the resulting smoothed distribution is a poor representation of the underlying distribution it is meant to represent. Secondly, such an approach would directly give us an indication of what parametric distribution to use as an approximation of the underlying ratio distribution.

### Supporting Information, section D – Project code

The project code can be found at http://folk.uio.no/trondr/simbies.

# References

Chib S, Greenberg E. Understanding the Metropolis-Hastings Algorithm. The Am. Stat. 2001:49:327–335.

Elston DA, Moss R, Boulinier T, Arrowsmith C, Lambin X. Analysis of aggregation, a worked example: numbers of ticks on red grouse chicks. Parasitology, 2001;122(5), 563-569.

Geyer CJ. . *Computing Science and Statistics*, Proceedings of the 23rd Symposium on the Interface, American Statistical Association, New York, 1991, p. 156.

Hastings WK. . Monte Carlo Sampling Methods Using Markov Chains and Their Applications. Biometrika. 1970:57(1): 97–109.

Jefferys WH, Berger JO. Ockham’s Razor and Bayesian Statistics, Am Sci. 1992:80:64–72.

Metropolis N, Rosenbluth AW, Rosenbluth MN, Teller AH, Teller E. Equations of State Calculations by Fast Computing Machines. J Chem Physics. 1953:21(6): 1087-1092.

Nielsen A, Dauber J, Kunin WE, Lamborn E, Jauker B, Moora M, et al. Pollinator community responses to the spatial population structure of wild plants: A pan-European approach. Basic and Applied Ecology. 2012:13:489-499.

Reitan T, Petersen-Øverleir A. Bayesian methods for estimating multi-segmented discharge rating curves. Stoch Env Res Risk Asses. 2009:23:627-642.

Taylor HM, Karlin S. An Introduction To Sctochastic Modeling. 3rd ed. San Diego: Academic Press; 1998. pp. 83-84.

# References

Figure S1: Histogram of ratios, z=x/y, where x and y are sampled from the negative binomial distribution. The total count for z=0 is not shown as it is much higher than the other ratios.

*Figure S2: Kernel smoothed density functions for ratio samples and a range of smoothing window size options (using the "density" function in R). Solid line: adjustment=1, short dashed line: adjustment=2.5, long dashed line: adjustment=10.*
